# Gel-Based NMR Method for Observing Submicrosecond Protein Dynamics at Atomic Resolution

**DOI:** 10.1021/acs.jpclett.6c01077

**Published:** 2026-05-12

**Authors:** Xinyao Xiang, Mamata Basnet, Mouzhe Xie, Lei Bruschweiler-Li, R. Brüschweiler

**Affiliations:** † Department of Chemistry and Biochemistry, 2647 The Ohio State University, Columbus, Ohio 43210, United States; ‡ School of Molecular Sciences, 7864 Arizona State University, , 551 E University Dr., Tempe, Arizona 85281, United States; § Department of Biological Chemistry and Pharmacology, The Ohio State University, Columbus, Ohio 43210, United States

## Abstract

NMR spectroscopy is uniquely suitable of observing functionally important protein motions at atomic resolution under near-physiological conditions in solution. Longitudinal and transverse spin relaxation experiments report about fast subnanosecond and low nanosecond motions, but they are insensitive to slower motions. The recently introduced nanoparticle assisted spin relaxation (NASR) method increases the observation window into the submicrosecond range by measuring the increase in transverse relaxation by the presence of silica nanoparticles. It is demonstrated here how a similar effect can be observed via the transverse relaxation enhancement Δ*R*_2_ due to the presence of polyacrylamide and agarose gel. While compressed or stretched polyacrylamide gels are commonly used in protein NMR for residual dipolar coupling measurements, the gel-induced transverse relaxation enhancement, for both compressed and uncompressed gel, directly provides complementary dynamics information with the change in *R*_2_ proportional to the site-specific model-free *S* ^2^ order parameter encompassing dynamics on the submicrosecond range. This generalized NASR approach is demonstrated for K-Ras and other proteins exhibiting internal dynamics with variable amplitudes on a wide range of time scales.

## Graphical abstract

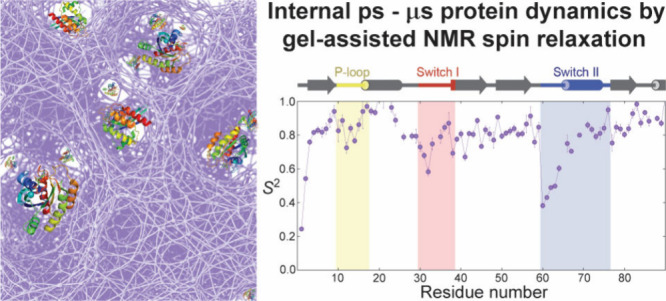

One of the key strengths of biomolecular NMR in solution is the ability to monitor protein dynamics processes at high spatial and temporal resolution under near-physiological conditions. Such information provides deep insights into protein functional processes, such as enzyme catalysis, molecular recognition, and allosteric signaling.^1,2^ Besides motional amplitudes, dynamics time scales play an important role too and they are often decisive whether a certain dynamics process is observable by a specific method. Among experimental NMR observables, heteronuclear spin relaxation parameters, especially longitudinal *R*_1_ (or 1/*T*_1_) rates, transverse *R*_2_ (or 1/*T*_2_) rates, and the heteronuclear Overhauser enhancement (hetNOE) of ^15^N spins play a special role as they allow a clean separation between structural and dynamics effects.^3^ They are dominated by the magnetic dipole–dipole interaction with their directly bonded hydrogen (^1^H) and the ^15^N chemical shielding anisotropy and, in the case of globular proteins, are most commonly analyzed via the model-free (MF) approach by converting them to a site-specific order parameter *S* ^2^, an internal correlation time τ_int_, and a global protein tumbling correlation time τ_P_.^4^ *S* ^2^ is a quantitative, site-specific scalar measure of the internal orientational motional restriction of the corresponding ^15^N–^1^H bond vector taking values between 1 (rigid) and 0 (fully flexible). An important limitation, however, is that the extracted *S* ^2^ values exclusively reflect internal motions faster than the overall tumbling τ_P_, i.e., motions on ps to low ns time scales. On the other hand, rotating frame relaxation experiments that are sensitive to chemical shift fluctuations, such as CPMG, are sensitive to μs–ms dynamics, thereby creating a blind spot for motions on intermediate ns−μs time scales.

In order to close this observation gap, we recently developed the nanoparticle-assisted spin relaxation method (NASR).^5^ NASR measures transverse ^15^N-*R*_2_ spin relaxation in the presence and absence of certain types of nanoparticles in the sample that are much larger than the protein molecules and, hence, tumble much more slowly than the free protein, typically on the high-ns to μs time scale. When the protein molecules transiently interact with the nanoparticles (NPs) they temporarily assume the slow nanoparticle tumbling correlation time (τ_NP_), thereby capturing dynamics information about slower time scale motions. For fast exchange between free and transiently bound protein, this information can be simply retrieved by measuring the differential transverse relaxation effect Δ*R*_2_ = *R*_2_^NP^ – *R*_2_^free^ = *cp*_b_τ_NP_ *S* ^2^, where *p*_b_ is the NP-bound protein population, and *S* ^2^ is the site-specific NASR order parameter, which reports on dynamics from ps to μs, thereby filling in the aforementioned observation gap.^5,9^ Prefactor *c* is a global constant affecting all ^15^N sites in an identical manner that depends on NMR interaction constants as well as the exchange rate *k*_ex_ between free and transiently bound protein and can be computed using the stochastic Liouville equation for the entire *k*_ex_ range^24^ or analytically for specific *k*_ex_ regimes.^7,8^ Importantly, slower time scale motions tend to occur in functionally critical protein regions, especially in loop regions that are directly involved in protein–protein and protein–ligand interactions.^5,9^

Nanoparticles well-suited for NASR applications of many different proteins are unmodified silica nanoparticles (SNPs) with a 20 nm diameter prepared as a colloidal dispersion and added to the protein NMR sample.^9^ Since many biomolecular NMR spectroscopists do not have hands-on experience with such synthetic nanoparticles, we explored alternatives that, instead of relying on NPs, are based on biomolecular sample preparation schemes that are more commonly used, while still exploiting the NASR principle to gain access to slow protein dynamics.

We demonstrate here how this is achieved by placing the proteins in a matrix composed of polyacrylamide gel (PAG) in aqueous solution.^10,11^ PAG is composed of a cross-linked network of acrylamide polymer chains forming cavities that accommodate protein molecules while still allowing them to diffuse translationally and rotationally, although in a slightly more restricted manner than free protein.^12^ PAG has been routinely used for many years in biomolecular NMR for the measurement of residual dipolar couplings (RDCs) by compressing or stretching the gel in the NMR tube, which causes the protein molecules, on average, to weakly align along the cylindrical symmetry axis of the NMR tube.^12,13^ The resulting RDCs of directly bonded ^15^N–^1^H spin pairs can be accurately measured providing detailed structural information about the proteins in solution.^14–16^

Here, we take advantage of differential NMR line broadening induced by the gel and show that the site-specific change in ^15^N-Δ*R*_2_ = *R*_2_^gel^ – *R*_2_^free^ is directly proportional to the generalized N–H order parameter *S* ^2^. Due to the restricted rotational diffusion imposed by the gel, *S* ^2^ is sensitive to sub-ns time scale motions as well as slower motions, thereby widening the observation window into the ns–sub-μs regime. Since this method is modeled after NASR, we term it “generalized NASR” or “gNASR”.

Uniformly ^15^N-labeled ubiquitin, Im7, and K-Ras were expressed and purified as described previously.^5,17^ The NMR samples of ubiquitin and Im7 were prepared by buffer exchange into 20 and 50 mM sodium phosphate buffer at pH 7.0, respectively. The K-Ras·GDP sample was prepared by buffer exchange into 20 mM HEPES buffer (pH 7.0), followed by the addition of 5 mM GDP, 5 mM MgCl_2_, and 2 mM TCEP.^17^ Each sample contained 5% (v/v) D_2_O, and protein concentrations were adjusted to 0.6–0.75 mM.

Polyacrylamide gels (7%) were prepared according to the protocol by Sass et al.^12^ The dried gel was placed in a 5 mm Shigemi tube, and an aqueous protein sample was added. The gel was allowed to swell in an unrestricted manner to 25 mm, forming a (near-)­isotropic protein solution, or had a fixed height of 18 mm, thereby compressing the gel vertically and causing the protein to weakly align along the cylindrical sample axis (see inset of Figure 1a). Reference spectra were obtained in the absence of polyacrylamide gel under otherwise identical protein concentrations and buffer conditions.

**Figure 1:**
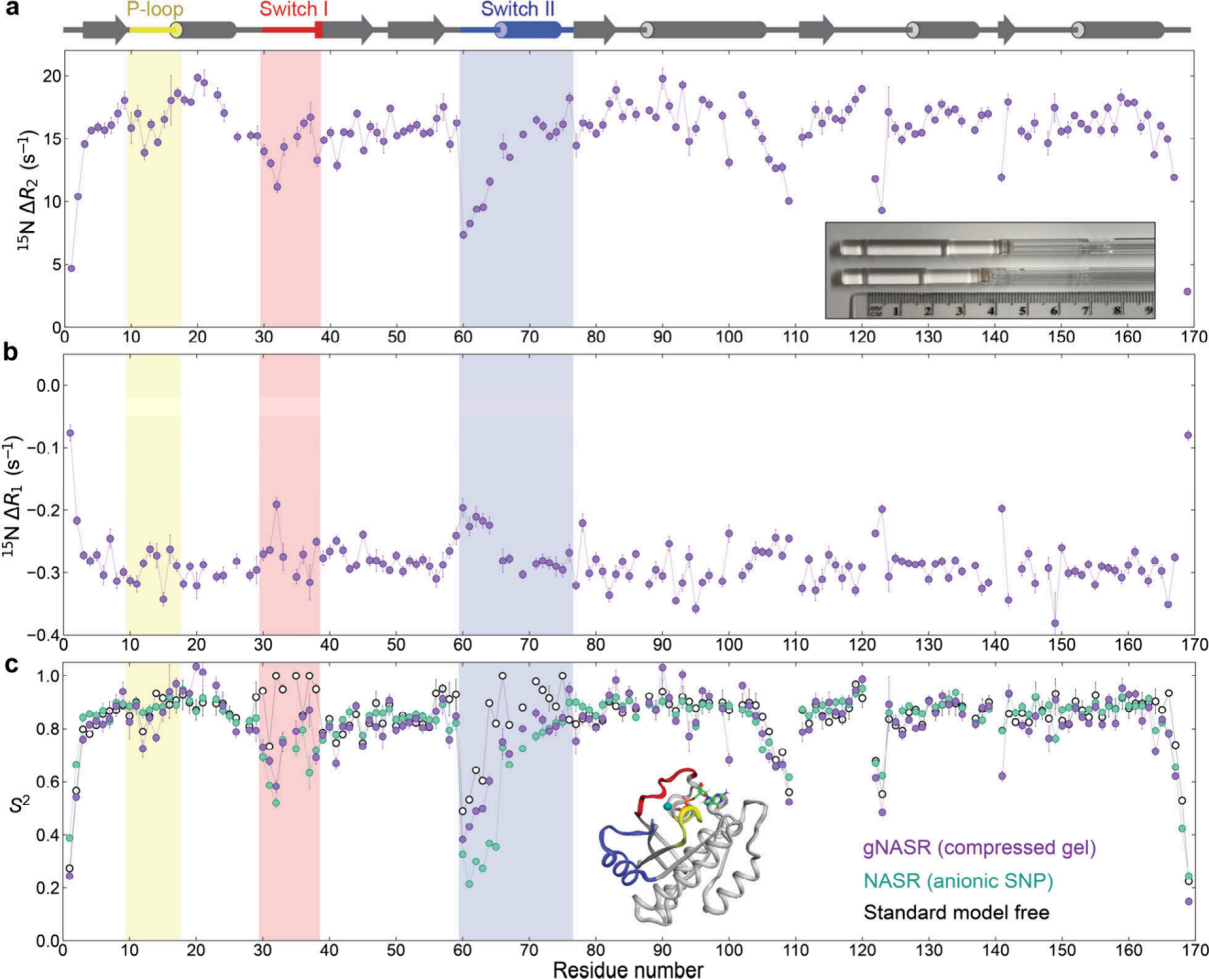
Backbone spin relaxation rates and order parameter profiles of K-Ras·GDP. The differences in backbone amide ^15^N transverse *R*_2_ and longitudinal *R*_1_ relaxation rates between the 7% PAG and free sample, Δ*R*_2_ = *R*_2_^gel^ −  *R*_2_^free^ and Δ*R*_1_ = *R*_1_^gel^ – *R*_1_^free^ profiles of K-Ras·GDP, are displayed in panels a and b (purple). The inset in (a) shows photos of vertically compressed, 1.8 cm long and uncompressed, 2.5 cm long gel samples. c) Generalized order parameters *S* ^2^ of the K-Ras·GDP backbone extracted from gNASR Δ*R*_2_ with 7% compressed PAG (purple), NASR with 20 nm anionic SNP (green), and standard model-free analysis (black open circles) are plotted against residue number. The P-loop, Switch I, and Switch II regions are highlighted in yellow, red, and blue, respectively. The inset shows an X-ray crystal structure of K-Ras·GDP (PDB 4OBE). The model-free *S* ^2^ were derived from the lean model-free approach with *R*_1_ and *R*_2_ relaxation rates of the free protein.^28^

NMR experiments were performed at 298 K on a Bruker AVANCE III spectrometer operating at 850 MHz ^1^H resonance frequency (19.97 T) equipped with a TCI cryoprobe. For each protein, ^1^H–^15^N HSQC spectra (Figure S1) and backbone amide ^15^N *R*_1_ and *R*_2_ spin relaxation rates in both the presence and absence of polyacrylamide gel were obtained from standard *R*_1_ and *R*_1ρ_ relaxation experiments^18–21^ with the recovery delay set to 2 s and a *R*_1ρ_ spin-lock field strength of 2000 Hz. The resulting *R*_1_ and *R*_1ρ_ relaxation rates were combined to obtain site-specific transverse *R*_2_ relaxation rates in standard fashion.^19^ The enhancements in ^15^N-*R*_2_ due to the presence of the gel were then determined for each resonance as Δ*R*_2_ = *R*_2_^gel^ – *R*_2_^free^. Δ*R*_1_ = *R*_1_^gel^ – *R*_1_^free^ was also determined providing complementary information about the effective viscosity increase experienced by the protein in the presence of the gel (*vide infra*). Furthermore, backbone amide ^15^N/^15^N–^1^H CSA/DD transverse cross-correlation rates η_*xy*_ were measured^6^ with a recovery delay of 1.5 s and a relaxation period T set between 50 and 80 ms. Analogous to Δ*R*_2_, η_*xy*_ increases in the presence of the gel with the enhancements determined for each ^15^N resonance as Δη_*xy*_ = η_*xy*_^gel^ – η_*xy*_^free^.

The experimentally determined changes Δ*R*_2_ can then be converted to *S* ^2^(gNASR) order parameters by uniform scaling with prefactor *d*, *S* ^2^(gNASR) = *d*·Δ*R*_2_, so that the most stable sections of secondary structural elements, namely the centers of the helices and β-sheets, have an average *S* ^2^(gNASR) = 0.85.^5^ The cross-correlation rates Δη_*xy*_ can be converted to cross-correlated *S*_η_^2^(gNASR) order parameters in a fully analogous manner.

Application of gNASR to K-Ras·GDP shows on average a large overall *R*_2_ increase ranging from 3 to 20 s^–1^ with the highest values of 15–20 s^–1^ found for the known regular secondary structures. By contrast, Δ*R*_1_ varies between −0.08 and −0.38 s^–1^ and are thus much smaller with negative values reflecting an increase of the effective viscosity experienced by the protein molecules inside PAG. The smallest Δ*R*_2_ values are exhibited by the tail and loop regions with the largest dip found for residues 60–64 at the beginning of Switch II, a part of K-Ras that is functionally important serving as a key target for anticancer drug design.^22^ Conversion of Δ*R*_2_ to *S* ^2^(gNASR) order parameters reveals close similarity with *S* ^2^(NASR) values using silica nanoparticles^17^ including Switch I, and loops around residues 105–110 and 116–125. For Switch II, the *S* ^2^(gNASR) values are slightly higher than *S* ^2^(NASR) but still lower than the standard MF *S* ^2^ values. These results show that, like NASR, gNASR is capable of extending the internal time scale observation window beyond the low-ns range detectable by standard MF.

The application of gNASR to Im7 and ubiquitin results in *S* ^2^ dynamics profiles shown in Figure 2 and Figure S2. The use of compressed gel (1.8 cm long PAG, purple) and uncompressed gel (2.5 cm PAG, pink) results in very similar profiles for both proteins. For Im7, the *S* ^2^(gNASR) profiles follow closely the standard MF *S* ^2^ profiles (black), except for Loop I (residues 27–31) where *S* ^2^(gNASR) reveals enhanced dynamics consistent with NASR (green) using anionic silica nanoparticles (SNPs). For ubiquitin, gNASR, NASR, and standard MF all yield very similar *S* ^2^ profiles suggesting the absence of any significant amounts of motions in the ns−μs range that could not be traced by standard MF. The close similarity between the *S* ^2^(gNASR) profiles of compressed and uncompressed gel shows that residual protein alignment, which is a prerequisite for RDC measurements, is nonessential for the manifestation of the gNASR effect. The slightly increased *S* ^2^(NASR) in the C-terminal tail of ubiquitin is due to weak electrostatic interactions between the basic arginine residues (R72, R74) and the anionic SNP surface.^23^

**Figure 2:**
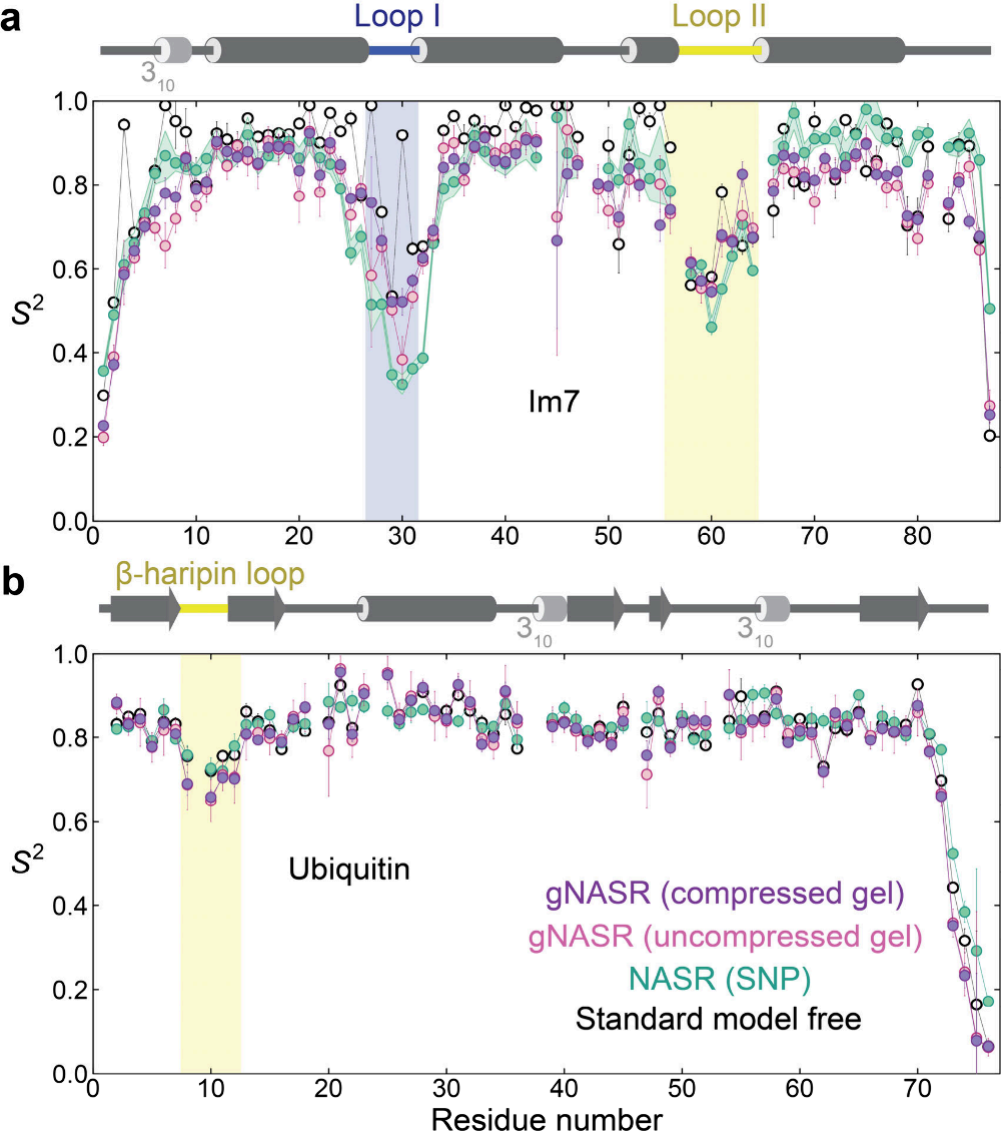
Backbone protein dynamics of Im7 and ubiquitin measured with gNASR. Generalized order parameter *S* ^2^ profiles as a function of the protein sequences derived from ^15^N relaxation rates using gNASR with compressed PAG (purple), gNASR with uncompressed PAG (pink), standard NASR with anionic (Im7) or neutral (ubiquitin) SNPs (green), and standard model-free analysis (black open circles) of (a) Im7 and (b) ubiquitin.

One of the characteristic properties of NASR is the absence of a measurable change in *R*_1_ by the presence of nanoparticles, which reflects unchanged viscosity in the presence of low levels of SNPs (typically in the low μM range). By contrast, *R*_1_ slightly decreases (i.e., the longitudinal relaxation time *T*_1_ increases) by ∼0.3 s^–1^ in the presence of PAG (Figure 1b), which reflects an increase of the microscopic viscosity experienced by the protein in the presence of the gel. A simple model for the protein tumbling behavior is depicted in Figure 3a, which assumes (i) an increase of the protein tumbling correlation τ_P_ in the presence of the gel and (ii) the transient, direct interaction of the protein with the gel walls during which the overall tumbling of the protein is suppressed (τ_P_ → *∞*). The latter effect is analogous to the (nonspecific) interaction of proteins with NPs, except that NPs undergo some, albeit very slow, rotational tumbling. The dependence of Δ*R*_2_(gNASR) relaxation rates (purple lines) on *S* ^2^, the protein internal motional time scale τ_int_, and *k*_ex_ is illustrated in Figure 3b calculated using the stochastic Liouville equation (SLE) formalism.^24^ Δ*R*_2_(gNASR) converges to the same maximum value independent of *S* ^2^ when τ_int_ approaches the low μs regime. The figure also shows the normalized ^15^N *R*_2_ (gray lines) for reference converging already when τ_int_ approaches τ_P_. The much slower convergence of Δ*R*_2_(gNASR) (purple lines) illustrates the extra sensitivity of gNASR to slow internal motions into the (sub-)­μs range, a time scale range not accessible by *R*_2_ (and *R*_1_) of the free protein in the absence of PAG. As for NASR, the contribution of conformational or chemical exchange *R*_ex_ to *R*_2_, which has to be explicitly accounted for in standard MF, is canceled out in Δ*R*_2_(gNASR), since *R*_ex_ is in excellent approximation independent of the presence of PAG.^5,17^

**Figure 3:**
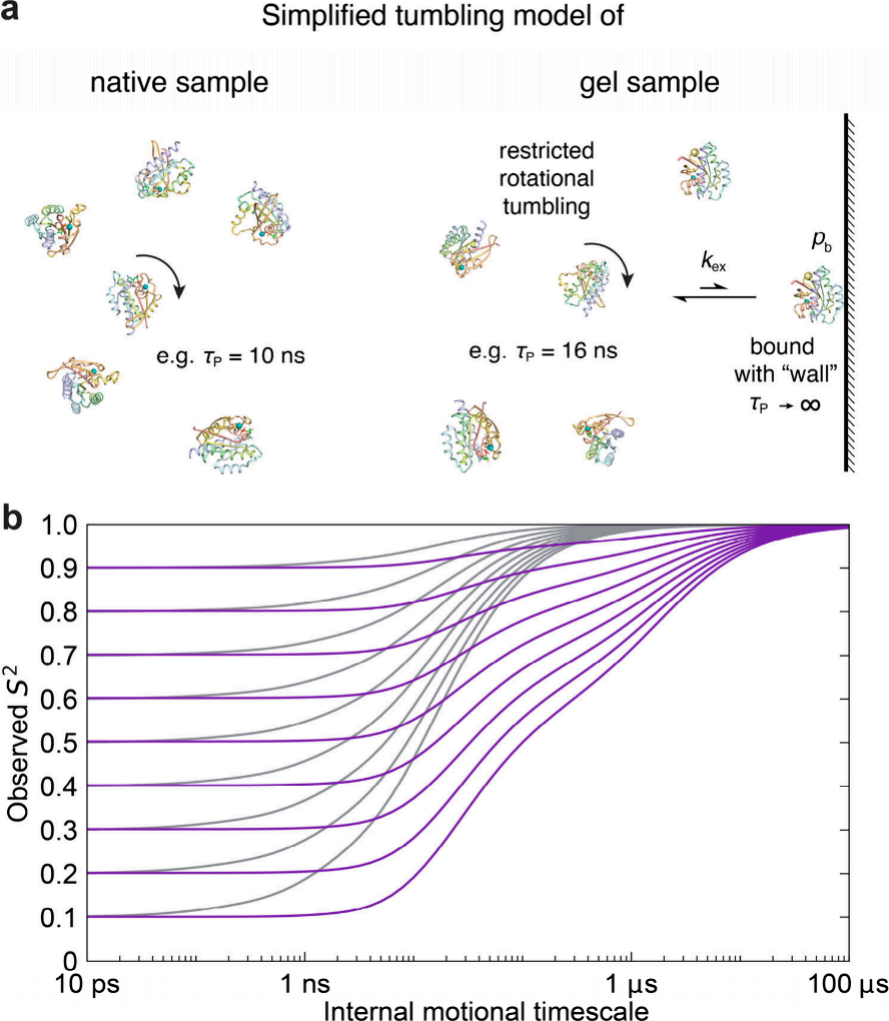
Simulations of the accessible time scale regime for protein internal motions of gNASR. a) Cartoon illustration of simplified rotational tumbling models of proteins in the absence and presence of gel used in the simulations. In the gel samples, proteins are assumed to have generally restricted and slower rotational tumbling. In addition, proteins are in fast exchange between the restricted tumbling state and a lowly populated, gel matrix-bound state, whereby the rotational tumbling is fully suppressed when bound to the gel matrix. b) Simulated dependence of normalized ^15^N *R*_2_ (gray) and gNASR Δ*R*_2_ (purple) rates on protein internal motional amplitude and time scale. The model parameters, which are representative for K-Ras, were chosen as follows: τ_P_ = 10 ns for proteins in the gel-free sample, τ_P_ = 16 ns for proteins in the gel sample in their unbound state, and τ_P_ → *∞* for proteins bound to the gel matrix. The bound population *p*_b_ is set to 0.25%, and *k*_ex_ to 5 × 10^5^. As a reference, the gray lines depict a situation when only restricted rotational diffusion is present (due to increased viscosity) with a tumbling correlation time of 16 ns. More simulations with different *p*_b_ and *k*_ex_ values are shown in Figure S5.

Instead of *R*_2_, one can also measure gNASR of the transverse dipole-CSA cross-correlated relaxation rates η_*xy*_,^6,19^ which reflect internal motions similar to *R*_2_ but are unaffected by conformational exchange contributions.^25^ The resulting Δη_*xy*_(gNASR) profiles for Im7 and ubiquitin show very good agreement with Δ*R*_2_(gNASR) (Figure S3). Notably, gels other than PAG can be used for gNASR as illustrated for ubiquitin in agarose gel (Figure S4).

As molecular dynamics (MD) computer simulations of protein systems now routinely reach into the microsecond range, their validation with respect to conformational dynamics using standard MF is inconclusive or incomplete due to the time scale mismatch.^26^ With their wide sensitivity range, experimental *S* ^2^(gNASR) values can provide useful benchmarks for the site-resolved validation of MD trajectories.

The gNASR method introduced here allows the observation of internal protein dynamics in a site-specific manner from ps–(sub-)­μs time scales by fractionally slowing down the effective overall tumbling of the protein due to a slight increase in viscosity along with nonspecific, transient interactions between the protein and the gel. It thereby significantly narrows or even closes the observation gap of traditional spin relaxation experiments. For the three proteins studied here, *S* ^2^(gNASR) are found to be either very similar to *S* ^2^(NASR) or slightly higher providing an upper limit of the true *S* ^2^ values. When using compressed PAG, the same sample can be used for both RDCs and gNASR measurements, thereby economizing sample preparation. Because gNASR takes advantage of a widely used sample preparation protocol, it should make this method readily deployable in many laboratories.

## Supplementary Material



## Data Availability

The relaxation rates have been deposited in the Dryad data repository.^27^

## Notes

Parameters used for spin relaxation rate calculations by SLE; figures of ^1^H–^15^N HSQC spectra in the absence and presence of PAG, ^15^N *R*_1_ and *R*_2_ relaxation rate profiles, Δη_*xy*_(gNASR) profiles, agarose gel-based gNASR profile, and simulated dependence of *S* ^2^(gNASR) on the protein internal motional time scale (PDF)
