# Supplementary material for: Gel-Based NMR Method for Observing Submicrosecond Protein Dynamics at Atomic Resolution
Source: J Phys Chem Lett. 2026 May 12;17(20):5765–9. doi: 10.1021/acs.jpclett.6c01077 (PMC13200236; doi:10.1021/acs.jpclett.6c01077)
Supplement: Supplementary file 1 [file jz6c01077_si_001.pdf]

Supporting Information

Gel-based NMR method for observing sub-microsecond protein dynamics at atomic resolution

Xinyao Xiang,[a,*] Mamata Basnet,[a] Mouzhe Xie,[b] Lei Bruschweiler-Li,[a] and R. Brüschweiler[a,c*]

[a] Department of Chemistry and Biochemistry, The Ohio State University, Columbus, Ohio 43210, United States.

[b] School of Molecular Sciences, Arizona State University, 551 E University Dr., Tempe, AZ 85281, United States

[c] Department of Biological Chemistry and Pharmacology, The Ohio State University, Columbus, Ohio 43210, United States.

*Corresponding authors:

Rafael Brüschweiler E-mail: bruschweiler.1@osu.edu

Xinyao Xiang E-Mail: xiang.197@osu.edu

Content

Spin relaxation rate calculations with SLE

Figure S1. Amide HSQC of K-Ras•GDP and ubiquitin in the absence and presence of gel

Figure S2. ^{15}N relaxation rates of Im7 and ubiquitin

Figure S3. Backbone dynamics from ^{15}N-$\Delta\eta_{xy}$ gNASR

Figure S4. Ubiquitin dynamics from agarose gel-based gNASR

Figure S5. Simulated dependence of gNASR-derived order parameters on the internal motional timescale of proteins

Spin relaxation rate calculations with SLE

The ^{15}N-R_2 relaxation rates in **Figure 3** and **Figure S5** were calculated by numerically solving the stochastic Liouville equation (SLE).[S1] The B_0-field strength was set to 850 MHz (19.96 T), the ^{1}H-^{15}N bond distance was set to 1.02 Å, and ^{15}N chemical shift anisotropy (CSA) $\Delta\sigma = -172$ ppm. The rotational tumbling times, bound population p_b, and free-bound exchange rate k_{ex} used are indicated in the figure captions. The internal motion was modeled with a two-site lattice jump for order parameters ≥ 0.3 and with a three-site jump for order parameters < 0.3.

References

(S1) Jameson, G.; Brüschweiler, R. NMR Spin Relaxation Theory of Biomolecules Undergoing Highly Asymmetric Exchange with Large Interaction Partners. *J. Chem. Theory Comput.* **2021**, *17* (4), 2374–2382. https://doi.org/10.1021/acs.jctc.1c00086.

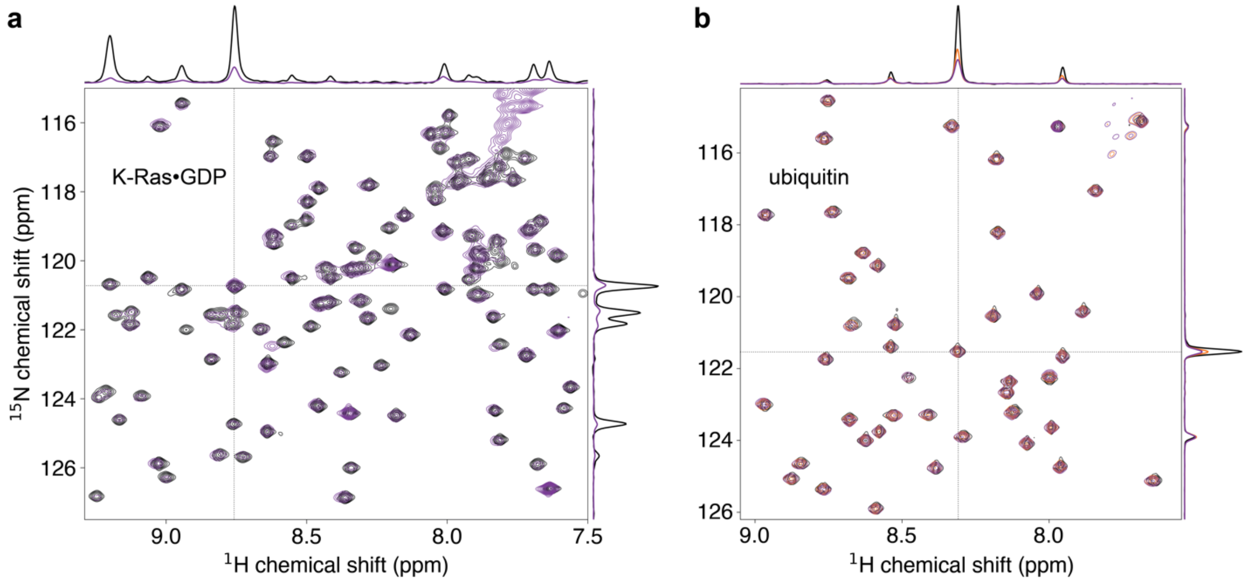

Figure S1. Amide HSQC of K-Ras•GDP and ubiquitin in the absence and presence of polyacrylamide gel (PAG). Selected ^{1}H-^{15}N HSQC spectral regions of (a) K-Ras•GDP and (b) ubiquitin in the absence (black) and presence of PAG are superimposed. Spectra of compressed PAG samples are shown in purple for both proteins, and the spectrum of ubiquitin in uncompressed PAG one is shown in orange (see also cross-sections). The 1D cross-sections taken at the positions indicated by the dashed lines are displayed at the top and on the right, highlighting the broadening of protein resonances in the gel samples.

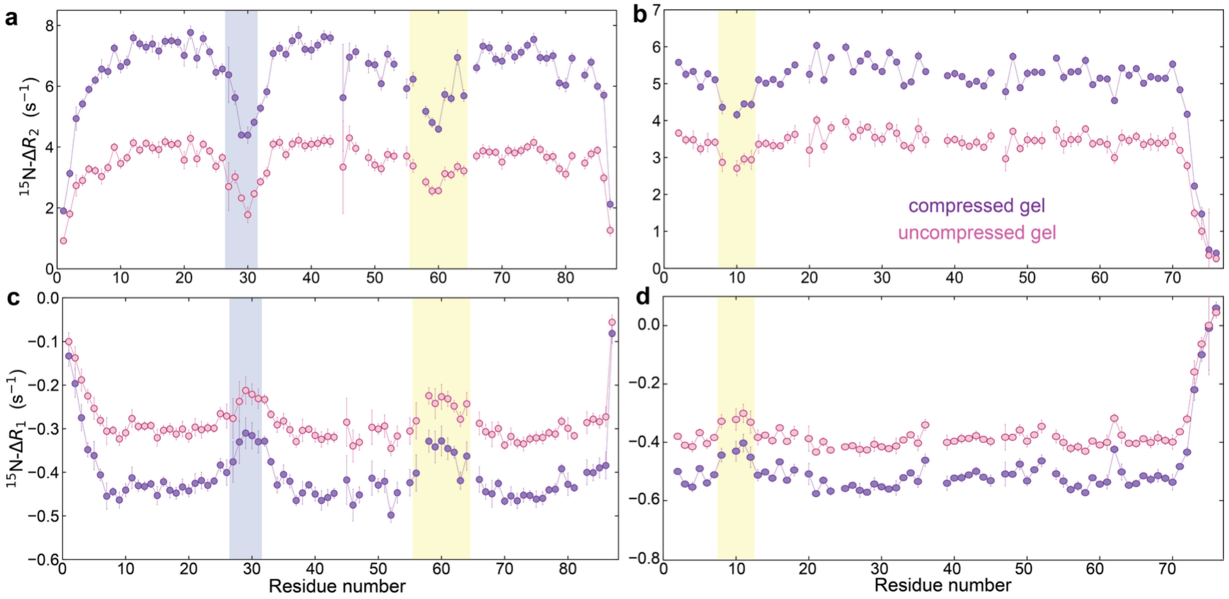

Figure S2. ^{15}N relaxation rates of Im7 and ubiquitin. The changes in protein ^{15}N backbone relaxation rates between gel and free samples are displayed as a function of residue number for (a,b) transverse ΔR_2 and (c,d) longitudinal ΔR_1 relaxation. Panels a,c show the profiles of protein Im7 and panels b,d of ubiquitin. The purple profiles are obtained with pairs of free and compressed PAG samples. The pink profiles belong to free and uncompressed PAG samples, and show overall smaller absolute ΔR_1 and ΔR_2 values compared to those observed for the compressed gel samples.

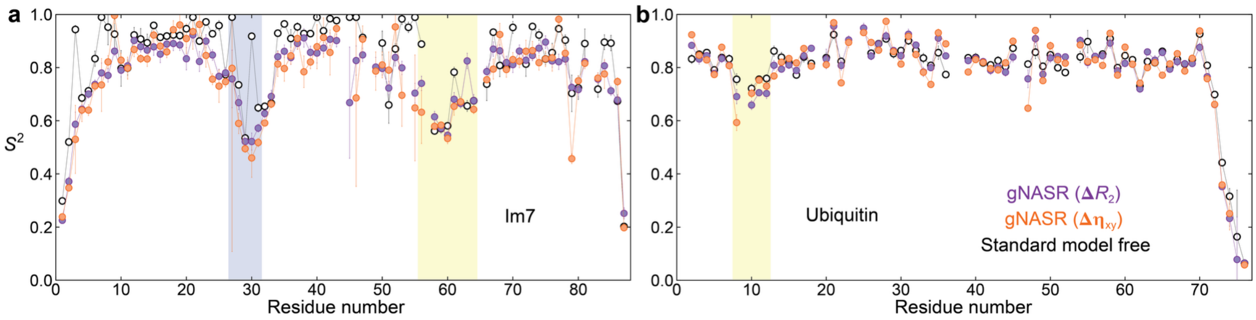

Figure S3. Backbone dynamics from ^{15}N-$\Delta\eta_{xy}$ gNASR. For both proteins (a) Im7 and (b) ubiquitin, the gNASR order parameters S^2 derived from ^{15}N-^{1}H DD/CSA transverse cross-correlation $\Delta\eta_{xy}$ (orange) agree well with those derived from ^{15}N transverse auto-correlated auto-relaxation ΔR_2 (purple) measured for pairs of gel-free and compressed PAG samples. The standard model-free order parameters are indicated in black open circles.

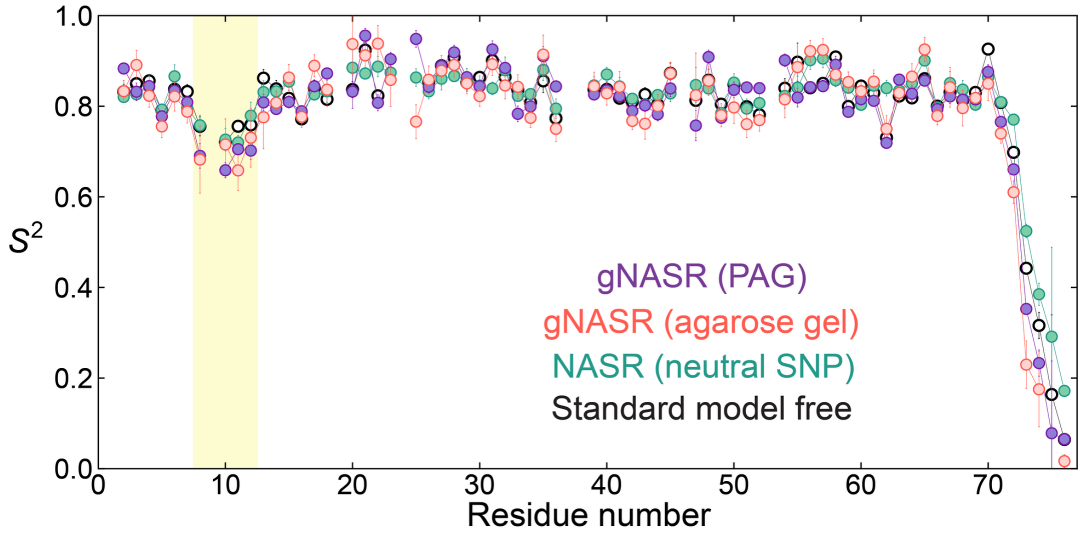

Figure S4. Ubiquitin dynamics from agarose gel-based gNASR. The ubiquitin gNASR order parameters derived from a pair of gel-free and 2% agarose gel samples are plotted in salmon as a function of residue number. The profile is in good agreement with gNASR results using compressed PAG (purple), NASR using neutral SNPs (green), and standard model-free (black open circles).

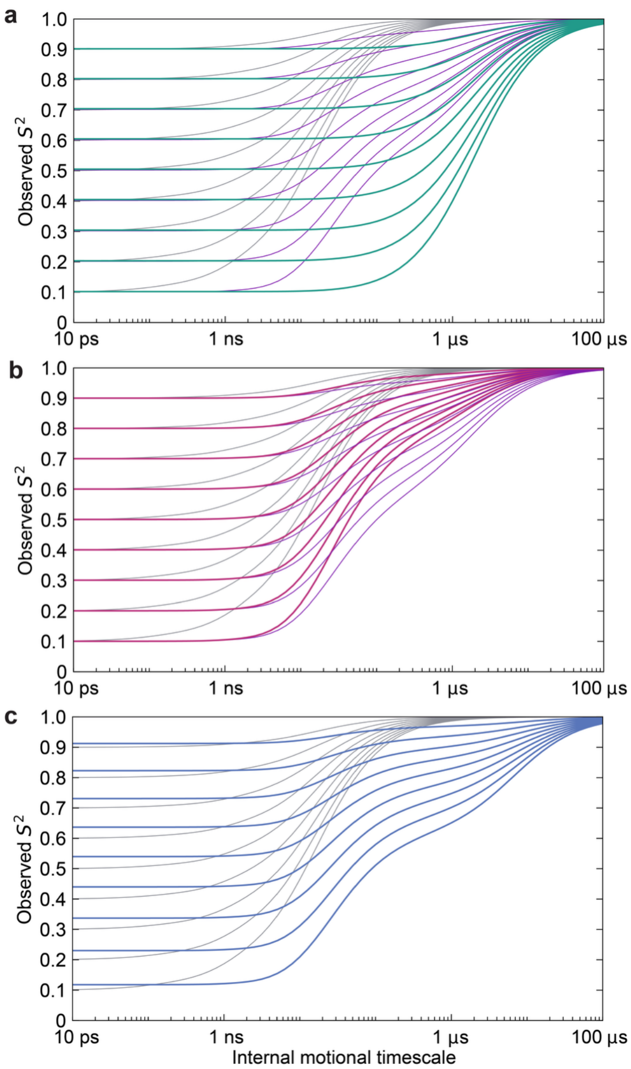

Figure S5. Simulated dependence of gNASR-derived order parameters on the internal motional timescale of proteins. The gray and purple lines, which are the normalized ^{15}N-R_2 and gNASR ΔR_2 values, are identical to those shown in **Figure 3** of the main text. Briefly, the gray lines were calculated using τ_P = 16 ns. For the purple lines, τ_P = 10 ns, 16 ns, and infinity were used for the gel-free sample, the restricted tumbling of the unbound state, and the bound state in the gel sample, respectively, with p_b = 0.25%, and k_{ex} = 5×10^5. In panel a, the green lines were calculated using the same p_b and k_{ex} values as the purple ones, but assuming the proteins in the unbound state in the gel sample do not have restricted rotational tumbling. In other words, only exchange with the gel matrix ("static wall") is considered, and the unbound state has the same τ_P = 10 ns as the free sample. In panel b, the pink lines were calculated with p_b reduced to 0.1% while all other parameters were kept the same as for the purple lines. In panel c, the blue lines show the simulations with k_{ex} = 1×10^5 corresponding to slower exchange compared to the purple ones. p_b was adjusted to 0.07% so that the absolute ΔR_2 value for a rigid N-H bond vector (S^2 = 1) remains the same as for the purple condition.
